# Clinicopathological features and impact of adjuvant chemotherapy on the long-term survival of patients with multiple gastric cancers: a propensity score matching analysis

**DOI:** 10.1186/s40880-019-0350-3

**Published:** 2019-02-11

**Authors:** Jian-Xian Lin, Zu-Kai Wang, Jian-Wei Xie, Jia-Bin Wang, Jun Lu, Qi-Yue Chen, Long-Long Cao, Mi Lin, Ru-Hong Tu, Ze-Ning Huang, Ju-Li Lin, Chao-Hui Zheng, Chang-Ming Huang, Ping Li

**Affiliations:** 1 Department of Gastric Surgery, Fujian Medical University Union Hospital, No. 29 Xinquan Road, Fuzhou, 350001 Fujian P. R. China; 2 Department of General Surgery, Fujian Medical University Union Hospital, Fuzhou, 350001 Fujian P. R. China; 3 Key Laboratory of Ministry of Education of Gastrointestinal Cancer, Fujian Medical University, Fuzhou, 350001 Fujian P. R. China

**Keywords:** Multiple gastric cancer, Solitary gastric cancer, Propensity score matching, Adjuvant, Chemotherapy, Prognosis, Eighth edition, American Joint Committee on Cancer

## Abstract

**Background:**

Little is known about the correlation between the clinicopathological features, postoperative treatment, and prognosis of multiple gastric cancers (MGCs). In this study, we aimed to investigate the correlation between these features and the impact of postoperative adjuvant chemotherapy on the long-term survival of patients with MGC.

**Methods:**

The clinical and pathological data of patients diagnosed with gastric adenocarcinoma who had radical gastrectomy from January 2007 to December 2016 were analyzed. Using propensity score matching, the prognostic differences, and the impact of postoperative adjuvant chemotherapy between those with MGC and solitary gastric cancers (SGC) were compared.

**Results:**

Among the 4107 patients investigated, the incidence of MGC was 3.2% (133/4107). Before matching, patients with MGC and SGC had disparities in the type of gastrectomy, pathological tumor stage (pT), pathological node stage (pN), and pathological tumor-node-metastasis stage (pTNM). After a 1:4 ratio matching, the clinical data of 133 cases of MGC and 532 cases of SGC were found to be comparable. The 5-year overall survival (OS) rate was 56.6% in the entire matched cohort, 48.1% in the MGC group, and 58.7% in the SGC group (*P* = 0.013). Multivariate analysis revealed that MGC, age, pT stage, pN stage, and adjuvant chemotherapy were independent predictors of OS (all *P* < 0.05). Stratified analyses demonstrated that for the cohort of advanced gastric cancer (AGC) patients who did not had adjuvant chemotherapy, the 5-year OS rate of advanced cases of MGC was inferior than that of SGC patients (34.0% vs. 46.1%, respectively; *P* = 0.025) but there were no significant difference in the 5-year OS rate between advanced MGC and SGC patients who had adjuvant chemotherapy (48.0% vs. 53.3%, respectively; *P* = 0.292). Further, we found that the 5-year OS rate of advanced MGC who had adjuvant chemotherapy was significantly higher than those who did not had adjuvant chemotherapy (48.0% vs. 34.0%, *P* = 0.026).

**Conclusions:**

Patients with advanced MGC was identified as having a poorer survival as to SGC patients, but the implementation of postoperative adjuvant chemotherapy showed that it had the potential to significantly improve the long-term prognoses of MGC patients.

## Introduction

Gastric cancer (GC) is the 4th most common malignant tumor and the 3rd leading cause of cancer-related mortality worldwide [1]. Multiple gastric cancers (MGCs) refer to gastric cancers with two or more cancerous lesions in the stomach [2]. With the advancement of diagnostic endoscopy techniques and detailed pathological examination of postoperative specimens, the detection of MGC has increased annually [3, 4]. The incidence of MGC reportedly accounts for 2%–8% of all gastric cancers [2–6]. Whether the clinical and pathological features of MGC are different from those of solitary gastric cancers (SGCs) remain controversial. Eom et al. found that MGCs were more likely to occur in elderly patients, males, individuals with a family history of cancer, cases of upper stomach tumors, and early gastric cancer patients [7]. However, Borie et al. found that there was no significant difference in the clinical data between MGC and SGC [8]. Furthermore, little is known about the impact of MGC on the survival of gastric cancer patients, and the effect of adjuvant chemotherapy on their long-term prognoses. Therefore, using a large cohort of GC patients, we aimed at comparing and correlating the differences in clinicopathological data and the impact of postoperative adjuvant chemotherapy on the long-term survival of patients with MGC as compared to those with SGC.

## Patients and methods

### Patients selection

The clinicopathological data of 4613 patients diagnosed with gastric adenocarcinoma who had radical gastrectomy at the Fujian Medical University Union Hospital (Fuzhou, Fujian, China) from January 2007 to December 2016 were retrospectively analyzed. The inclusion criteria were as follows: (1) preoperatively confirmed diagnosis of GC; (2) no evidence of distant metastasis before surgery; (3) radical gastrectomy (R0 resection); and (4) no records of other additional adjuvant treatments other than adjuvant chemotherapy. The exclusion criteria were as follows: (1) a history of previous malignancy (*n* = 74); (2) a history of previous gastric surgery (*n* = 77); (3) treatment with neoadjuvant chemotherapy (*n* = 140); and (4) pathological T4b stage tumor (*n* = 95); and (5) incomplete clinical and pathological data (*n* = 120). All patients’ clinical and pathological data were collected using the Gastric Cancer Clinical Data Management and Analysis System (Lisheng, Guangzhou, Guangdong, China) [9]. The surgical approach was performed according to the guidelines of the Japanese Society of Gastric Cancer Association [10]. Each resected specimen was histologically carefully examined by an expert pathologist with an experience of more than 5 years. The histological type, number of examined lymph nodes (eLNs), and number of lymph node metastases were recorded from the postoperative pathological report. This study was approved by the ethics committee of Fujian Medical University Union Hospital and was allocated with the IRB number 2018KY040. Each subject provided signed informed consent before participating to this study.

### Definition of MGC

The definition of MGC complied with the criteria proposed by Moertel et al. [2], which are as follows: (1) each lesion must be pathologically proven to be malignant; (2) all lesions must be separated by areas of microscopically normal gastric wall; and (3) the possibility that one of the lesion represents a local extension of a metastatic tumor must be ruled out beyond reasonable doubt. When multiple gastric lesions infiltrated different depths of the stomach wall, the cancer was defined and staged according to the more advanced lesion (depth of invasion) or according to the larger lesion if the depth of invasion was the same. When multiple gastric lesions had different histological types, the cancer was classified according to the less differentiated type.

### Variables and definitions

Variables included in this study were age, sex, body mass index (BMI), family history, tobacco and alcohol use, the American Society of Anesthesiologists (ASA) score, comorbidities, clinical tumor stage (cT stage), clinical node stage (cN stage), surgical approach, type of gastrectomy, number of eLNs, pathological tumor stage (pT stage), pathological node stage (pN stage), pathological tumor-node-metastasis stage (pTNM stage), tumor size, tumor histological type, postoperative complications, and adjuvant chemotherapy. The patients’ BMI were classified into these following categories: < 18.5, 18.5–24.9 and ≥ 25 kg/m^2^, based on the World Health Organization (WHO) classification standards. Family history for the first- and second-degree relatives was queried and was categorized as absence of family history, family history of GC, and family history of other malignancy. The comorbidities analyzed in this study comprised of hypertension, diabetic mellitus, heart disease (coronary atherosclerotic heart disease, arrhythmia, etc.), pulmonary disease (chronic obstructive pulmonary disease, asthma, pneumonia, etc.), central nervous system disease (cerebrovascular disease, neurodegenerative disease, etc.), liver disease (cirrhosis, hepatitis, etc.), renal disease (chronic kidney disease, nephritis, etc.), anemia, hypoalbuminemia, and hyperthyroidism. The postoperative pathological stage of the tumor was determined according to the eighth edition of the American Joint Committee on Cancer (AJCC) and the Union for International Cancer Control (UICC) staging manual [11]. The type of gastrectomy was categorized as total gastrectomy, distal gastrectomy, and proximal gastrectomy. The number of eLNs was grouped as ≤ 15 and > 15 eLNs according to the 4th version of the Japanese gastric cancer treatment guidelines [10]. Based on the Japanese classification of gastric carcinoma, early GC was defined as lesions confined to the mucosa or submucosa, regardless to the presence of regional lymph node metastasis, while advanced GC was defined as T2-T4 carcinoma without distant metastasis [12]. Most advanced GC patients were recommended to receive 6–8 cycles of oxaliplatin (Sanofi, Paris, France) plus capecitabine (Roche, Basel, Switzerland) (XELOX regimen) or oxaliplatin plus S-1 (Taiho, Tokyo, Japan) (SOX regimen) chemotherapy regimen [13, 14]. All treatment cycles were administered every 3 weeks. Oxaliplatin was infused intravenously for 2 h on day 1 at a dose of 130 mg/m^2^. Capecitabine/S-1 was administered orally twice daily from day 1 to 14 (dosage: capecitabine, 2000 mg/m^2^/day; S-1, 80–120 mg/m^2^/day), followed by 1-week rest; after which the next cycle was resumed.

### Follow-up

Follow-up evaluation after surgery generally consisted of clinic visits, serologic chemistry profiles, and computed tomography (CT) scans repeated every 3–6 months for the first 2 years then every 6–12 months for the following 3–5 years, and annually afterwards. The overall survival (OS) time was recorded as from the date of surgery to the last follow-up date, date of death, or date until the end of follow-up (such as loss to follow-up or death due to other diseases). The last date of follow-up was June 2017.

### Statistical analysis

Categorical variables were analyzed using the Chi square test or Fisher’s exact test, whereas continuous variables were analyzed using the student’s *t*-test or the Mann–Whitney U test. The propensity score matching ratio was set to 1:4 ratio to minimize the differences between MGC and SGC due to age, sex, BMI, ASA score, comorbidities, clinical stage, type of gastrectomy, and pathological stage with the nearest neighbor method using R software (version 3.4.2, “MatchIt” and “Foreign” packages, http://www.r-project.org) [15, 16]. The Kaplan–Meier method was used to calculate the time-dependent survival probabilities. The log-rank test was applied for statistical comparisons between survival curves. Univariate and multivariate analysis was performed using the Cox proportional hazards model, and all significant variables (*P* < 0.05) in the univariate analysis were computed into the multivariate analysis to identify the independent prognostic factors. Statistical analyses were performed using the IBM SPSS software (version 22.0, Chicago, IL, USA) and the R software, all statistical tests were two-sided, and a *P* value < 0.05 was considered statistically significant.

## Results

### Incidence and distribution of the MGC within the stomach

**Fig. 1:**
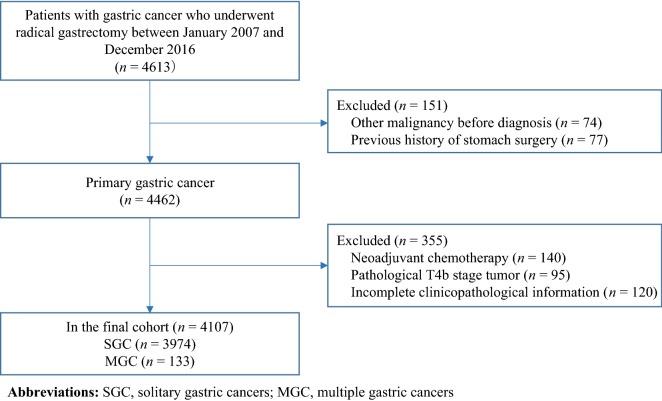
Case screening process of this study. *SGC* solitary gastric cancer, *MGC* multiple gastric cancer

**Fig. 2:**
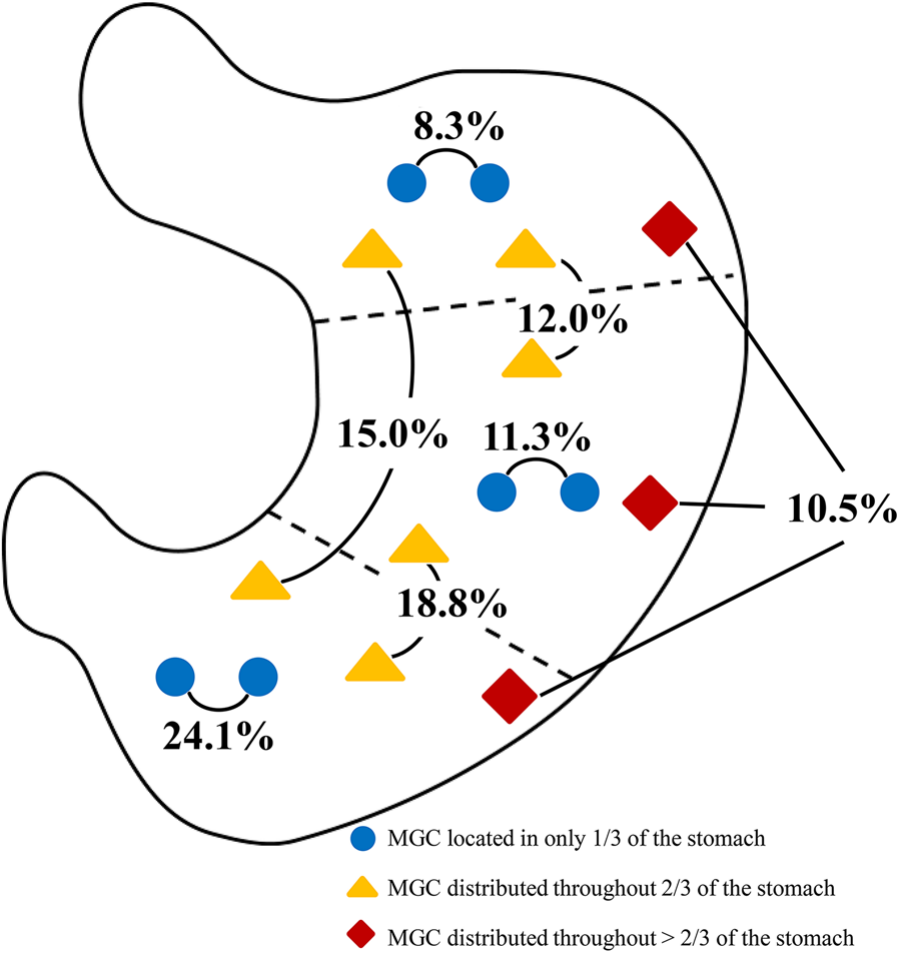
Distributions of the multiple gastric cancers (MGC). There were 43.7% of the MGC located in only one-third of the stomach (blue circle), 45.8% distributed throughout two-thirds of the stomach (yellow triangle), and the other 10.5% covering more than two third of the stomach (red rhombus)

A total of 4107 patients were included in this study, comprising of 133 (3.2%) patients with MGC and 3974 (96.8%) patients with SGC (Fig. 1). The distribution of MGC in the stomach is shown in Fig. 2. The MGC lesions of 11 (8.3%) patients were located in the upper third of the stomach, 15 (11.3%) in the middle third, and 32 (24.1%) in the lower third of the stomach. The MGC lesions were identified in more than one locations, namely in the upper and middle third, the upper and lower third, and the middle and lower third of the stomach in 16 (12.0%), 20 (15.0%), and 25 (18.8%) patients, respectively. Further, in 14 (10.5%) patients, the lesions were simultaneously identified in the upper, middle, and lower third of the stomach.

### Comparison of the clinical and pathological data between the MGC and SGC groups before and after propensity score matching

Before matching, there were statistically significant differences in the type of gastrectomy (*P* < 0.001), pT stage (*P* = 0.033), pN stage (*P* = 0.035) and pTNM stage (*P* = 0.041) between the two groups, but no significant differences were observed in the preoperative data, namely, age, sex, BMI, family history, tobacco and alcohol use, ASA score, comorbidities, cT stage, or cN stage (all *P* ≥ 0.05) (Tables 1 and 2).

**Table 1:** Preoperative clinical characteristics of SGC and MGC in the entire cohort (*n* = 4107) and after propensity score matching (*n* = 665)

Parameters	Entire cohort (before matching)		*P* value	Propensity score matched cohort		*P* value
	SGC, *n* (%)	MGC, *n* (%)		SGC, *n* (%)	MGC, *n* (%)	
Age (years)			0.141			0.968
≤ 60	1871 (47.1)	54 (40.6)		215 (40.4)	54 (40.6)	
> 60	2103 (52.9)	79 (59.4)		317 (59.6)	79 (59.4)	
Sex			0.166			0.886
Male	2924 (73.6)	105 (78.9)		423 (79.5)	105 (78.9)	
Female	1050 (26.4)	28 (21.1)		109 (20.5)	28 (21.1)	
BMI (kg/m^2^)			0.467			0.947
BMI < 18.5	385 (9.7)	13 (9.8)		57 (10.7)	13 (9.8)	
18.5 ≤ BMI < 25	2921 (73.5)	103 (77.4)		406 (76.3)	103 (77.4)	
BMI ≥ 25	668 (16.8)	17 (12.8)		69 (13.0)	17 (12.8)	
Family history^a^			0.273			0.269
Absent	3736 (94.0)	126 (94.7)		518 (97.3)	126 (94.7)	
Gastric cancer	128 (3.2)	6 (4.5)		11 (2.1)	6 (4.5)	
Other malignancy	110 (2.8)	1 (0.8)		3 (0.6)	1 (0.8)	
Tobacco use			0.754			0.965
No	2849 (71.7)	97 (72.9)		389 (73.1)	97 (72.9)	
Yes	1125 (28.3)	36 (27.1)		143 (26.9)	36 (27.1)	
Alcohol use			0.410			0.532
No	3657 (92.0)	125 (94.0)		507 (95.3)	125 (94.0)	
Yes	317 (8.0)	8 (6.0)		25 (4.7)	8 (6.0)	
ASA score			0.168			0.641
I	1954 (49.2)	57 (42.9)		241 (45.3)	57 (42.8)	
II	1818 (45.7)	71 (53.4)		269 (50.6)	71 (53.4)	
III	193 (4.9)	4 (3.0)		21 (3.9)	4 (3.0)	
IV	9 (0.2)	1 (0.8)		1 (0.2)	1 (0.8)	
Comorbidities^b^			0.464			0.715
Absent	1226 (30.9)	88 (66.2)		343 (64.5)	88 (66.2)	
Present	2748 (69.1)	45 (33.8)		189 (35.5)	45 (33.8)	
cT stage			0.920			0.785
T1–T3	2074 (52.2)	70 (52.6)		287 (53.9)	70 (52.6)	
T4	1900 (47.8)	63 (47.4)		245 (46.1)	63 (47.4)	
cN stage			0.050			0.806
N0	1653 (41.6)	44 (33.1)		182 (34.2)	44 (33.1)	
N+	2321 (58.4)	89 (66.9)		350 (65.8)	89 (66.9)	

*SGC* solitary gastric cancers, *MGC* multiple gastric cancers, *BMI* body mass index, *ASA* American Society of Anesthesiologists, *cT stage* clinical tumor stage, *cN stage* clinical node stage, *cN0* clinical node-negative, *cN+* clinical node-positive

^a^*Family history* presence of cancer within the patients’ first- and second- degree relatives

^b^*Comorbidities* including hypertension, diabetic mellitus, heart disease (coronary atherosclerotic heart disease, arrhythmia, etc.), pulmonary disease (chronic obstructive pulmonary disease, asthma, pneumonia, etc.), central nervous system disease (cerebrovascular disease, neurodegenerative disease, etc.), liver disease (cirrhosis, hepatitis, etc.), renal disease (chronic kidney disease, nephritis, etc.), anemia, hypoalbuminemia, and hyperthyroidism

**Table 2:** Surgical and postoperative pathological characteristics, and treatment of the SGC and MGC of the entire cohort (*n* = 4107) and after propensity score matching (*n* = 665)

Parameters	Entire cohort (before matching)		*P* value	Propensity score matched cohort		*P* value
	SGC, *n* (%)	MGC, *n* (%)		SGC, *n* (%)	MGC, *n* (%)	
Surgical approach			0.414			0.959
Open	802 (20.2)	23 (17.3)		93 (17.5)	23 (17.3)	
Laparoscopic	3172 (79.8)	110 (82.7)		439 (82.5)	110 (82.7)	
Type of gastrectomy			< 0.001			1.000
Total	2129 (53.6)	106 (79.7)		424 (79.7)	106 (79.7)	
Distal	1782 (44.8)	27 (20.3)		108 (20.3)	27 (20.3)	
Proximal	63 (1.6)	0 (0.0)		0 (0.0)	0 (0.0)	
Number of eLNs			0.547			0.514
≤ 15	161 (4.1)	4 (3.0)		11 (2.1)	4 (3.0)	
> 15	3813 (95.9)	129 (97.0)		521 (97.9)	129 (97.0)	
pT stage			0.033			0.706
T1	983 (24.7)	20 (15.0)		98 (18.4)	20 (15.0)	
T2	446 (11.2)	17 (12.8)		54 (10.2)	17 (12.8)	
T3	1165 (29.3)	37 (27.8)		144 (27.1)	37 (27.8)	
T4a	1380 (34.7)	59 (44.4)		236 (44.4)	59 (44.4)	
pN stage			0.035			0.909
N0	1442 (36.3)	37 (27.8)		157 (29.5)	37 (27.8)	
N1	601 (15.1)	16 (12.0)		73 (13.7)	16 (12.0)	
N2	680 (17.1)	23 (17.3)		86 (16.2)	23 (17.3)	
N3	1251 (31.5)	57 (42.9)		216 (40.6)	57 (42.9)	
pTNM stage			0.041			0.430
Stage I	1143 (28.8)	25 (18.8)		123 (23.1)	25 (18.8)	
Stage II	875 (22.0)	32 (24.1)		107 (20.1)	32 (24.1)	
Stage III	1956 (49.2)	76 (57.1)		302 (56.8)	76 (57.1)	
Tumor size			0.222			0.843
≤ 5 cm	2652 (66.7)	82 (61.7)		323 (60.7)	82 (61.7)	
> 5 cm	1322 (33.3)	51 (38.3)		209 (39.3)	51 (38.3)	
Tumor histological type			0.640			0.860
Differentiated	1150 (28.9)	36 (27.1)		140 (26.3)	36 (27.1)	
Undifferentiated	2824 (71.1)	97 (72.9)		392 (73.7)	97 (72.9)	
Postoperative complications			0.242			0.481
Absent	3182 (80.1)	101 (75.9)		419 (78.8)	101 (75.9)	
Present	792 (19.9)	32 (24.1)		113 (21.2)	32 (24.1)	
Adjuvant chemotherapy^a^			0.069			0.852
No	1263 (42.2)	38 (33.6)		150 (34.6)	38 (33.6)	
Yes	1728 (57.8)	75 (66.4)		284 (65.4)	75 (66.4)	

*SGC* solitary gastric cancers, *MGC* multiple gastric cancers, *eLNs* examined lymph nodes, *pT stage* pathological tumor stage, *pN stage* pathological node stage, *pTNM stage* pathological tumor-node-metastasis stage

^a^*Adjuvant chemotherapy* only patients with advanced gastric cancer were analyzed, which included 3104 patients before matching and 547 after matching

After the propensity score matching ratio was set to 1:4, the clinicopathologic data of 133 patients in the MGC group were found comparable with 532 patients in the SGC group (*P* > 0.05 between all variables) (Tables 1 and 2).

### Impact of MGC on 5-year OS

**Fig. 3:**
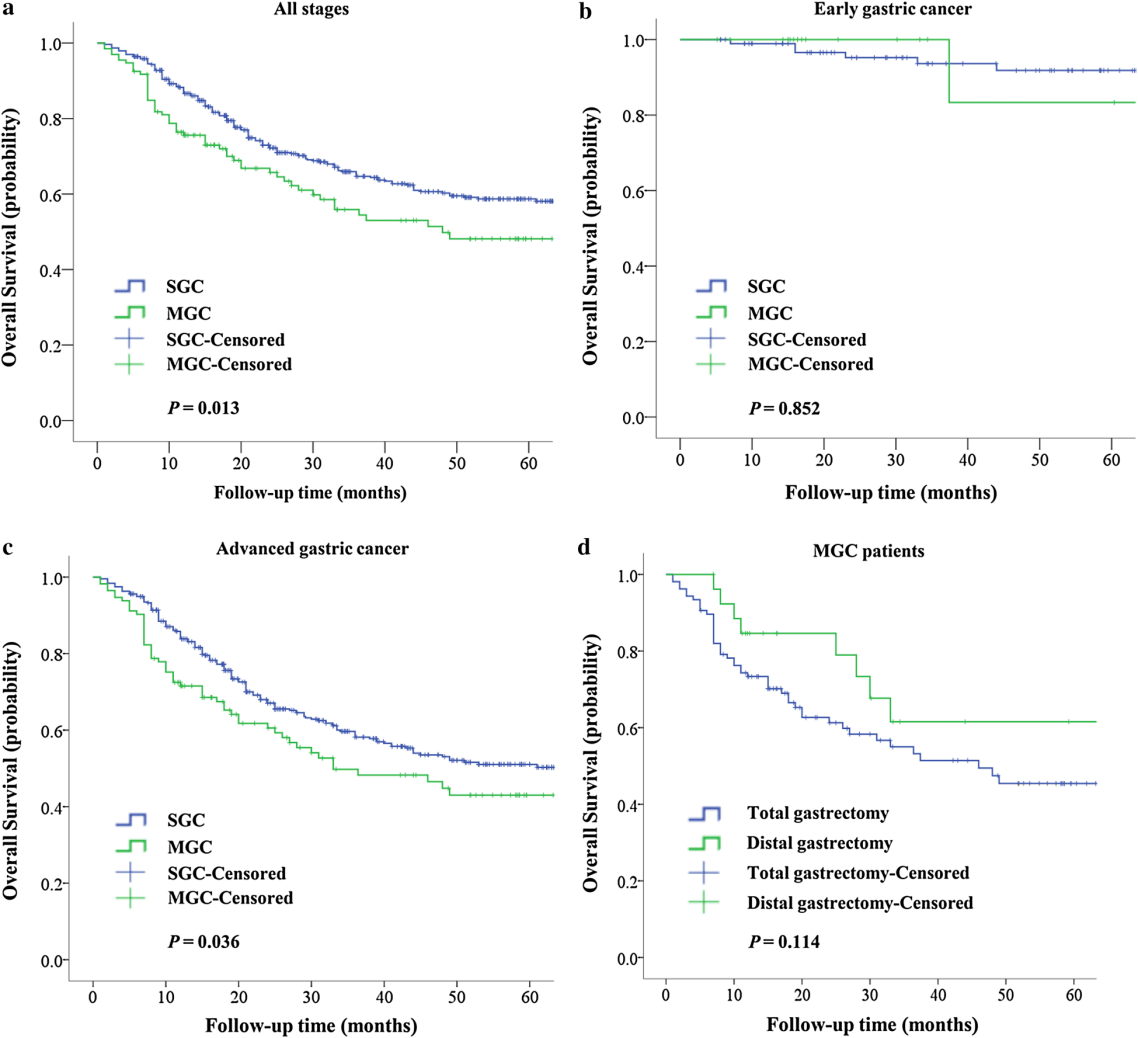
Impact of MGC on OS. **a** In all stages (early and advanced) of gastric cancer. The 5-year OS rate was 56.6% for the entire study cohort. For the MGC and SGC groups, the 5-year OS rates were 48.1% and 58.7%, respectively (*P* = 0.013). **b** In the early stage. The 5-year OS rate was 91.2%. For the MGC and SGC groups, the 5-year OS rates were 83.3% and 91.8%, respectively (*P* = 0.852). **c** In the advanced stage. The 5-year OS rate was 49.4%. For the MGC and SGC groups, the 5-year OS rates were 43.0% and 51.1%, respectively (*P* = 0.036). **d** The 5-year OS rates of MGC patients who underwent total gastrectomy and distal gastrectomy were 45.4% and 61.5%, respectively (*P* = 0.114). *MGC* multiple gastric cancers, *SGC* solitary gastric cancers, *OS* overall survival

After matching, the median follow-up time of the entire matched cohort was 31 months (range 1–127 months), and the 5-year OS rate was 56.6%. For the MGC and SGC groups, the 5-year OS rates were 48.1% and 58.7%, respectively (*P* = 0.013, Fig. 3a).

After stratification based on the stage of the tumor, the 5-year OS rate of the early GC cohort was 91.2%, and no statistically significant difference in the 5-year OS rates between the early MGC (5-year OS, 83.3%) and SGC (5-year OS, 91.8%) were found (*P* = 0.852, Fig. 3b). The 5-year OS rate for the cohort of advanced GC patients was 49.4%, among which the 5-year OS rates of the advanced MGC and SGC group were 43.0% and 51.1% respectively, and significant differences in survival between the two groups were observed (*P* = 0.036, Fig. 3c). When stratified by the types of gastrectomy, the 5-year OS rates of MGC patients who underwent total gastrectomy and distal gastrectomy were 45.4% and 61.5%, respectively, but no significant difference in 5-year OS was found (*P* = 0.114, Fig. 3d).

### Univariate and multivariate analysis for OS

Univariate analysis showed that MGC (*P* = 0.014), age (*P* = 0.029), cT stage (*P* < 0.001), cN stage (*P* < 0.001), surgical approach (*P* < 0.001), type of gastrectomy (*P* < 0.001), pT stage (*P* < 0.001), pN stage (*P* < 0.001), tumor size (*P* < 0.001), histological subtype (*P* = 0.009), and adjuvant chemotherapy (*P* = 0.032) were correlated with OS. However, on multivariate analysis only MGC (*P* = 0.039), age (*P* = 0.043), pT stage (*P* < 0.001), pN stage (*P* < 0.001), and adjuvant chemotherapy (*P* = 0.014) were identified as independent risk factors for OS (Table 3).

**Table 3:** Univariate and multivariate analysis to determine the prognostic factors for the overall survival in the matched cohort

Parameters	Univariate analysis	*P* value	Multivariate analysis	*P* value
	HR (95% CI)		HR (95% CI)	
MGC				
Absent	Ref		Ref	
Present	1.456 (1.079–1.964)	0.014	1.378 (1.016–1.870)	0.039
Age (years)				
≤ 60	Ref		Ref	
> 60	1.343 (1.03–1.751)	0.029	1.319 (1.009–1.726)	0.043
cT stage				
T1–T3	Ref		Ref	
T4	2.862 (2.181–3.755)	< 0.001	0.931 (0.663–1.307)	0.681
cN stage				
N0	Ref		Ref	
N+	2.592 (1.882–3.568)	< 0.001	1.085 (0.762–1.545)	0.652
Surgical approach				
Open	Ref		Ref	
Laparoscopic	0.611 (0.458–0.815)	< 0.001	0.777 (0.577–1.048)	0.098
Type of gastrectomy				
Total	Ref		Ref	
Distal	0.479 (0.328–0.699)	< 0.001	0.794 (0.530–1.187)	0.261
pT stage		< 0.001		< 0.001
T1	Ref		Ref	
T2	2.57 (0.996–6.629)	0.051	2.011 (0.760–5.324)	0.159
T3	5.776 (2.620–12.732)	< 0.001	2.996 (1.231–7.292)	0.016
T4a	13.604 (6.384–28.989)	< 0.001	4.944 (1.974–12.383)	0.001
pN stage		< 0.001		< 0.001
N0	Ref		Ref	
N1	2.107 (1.122–3.955)	0.02	1.413 (0.704–2.835)	0.330
N2	3.254 (1.887–5.610)	< 0.001	1.667 (0.878–3.164)	0.118
N3	8.778 (5.563–13.85)	< 0.001	3.557 (1.98–6.392)	< 0.001
Tumor size				
≤ 5 cm	Ref		Ref	
> 5 cm	2.986 (2.304–3.871)	< 0.001	1.314 (0.982–1.758)	0.066
Histological subtype				
Differentiated	Ref		Ref	
Undifferentiated	1.564 (1.120–2.183)	0.009	1.077 (0.765–1.516)	0.672
Adjuvant chemotherapy^a^				
No	Ref		Ref	
Yes	1.335 (1.025–1.739)	0.032	0.706 (0.535–0.932)	0.014

*HR* hazard ratio, *CI* confidence interval, *MGC* multiple gastric cancers, *cT stage* clinical tumor stage, *cN stage* clinical node stage, *cN0* clinical node-negative, *cN+* clinical node-positive, *pT stage* pathological tumor stage, *pN stage* pathological node stage, *Ref* reference

^a^*Adjuvant chemotherapy* only the matched 547 patients with advanced gastric cancer were analyzed

### Impact of chemotherapy on the survival of advanced MGC patients

**Fig. 4:**
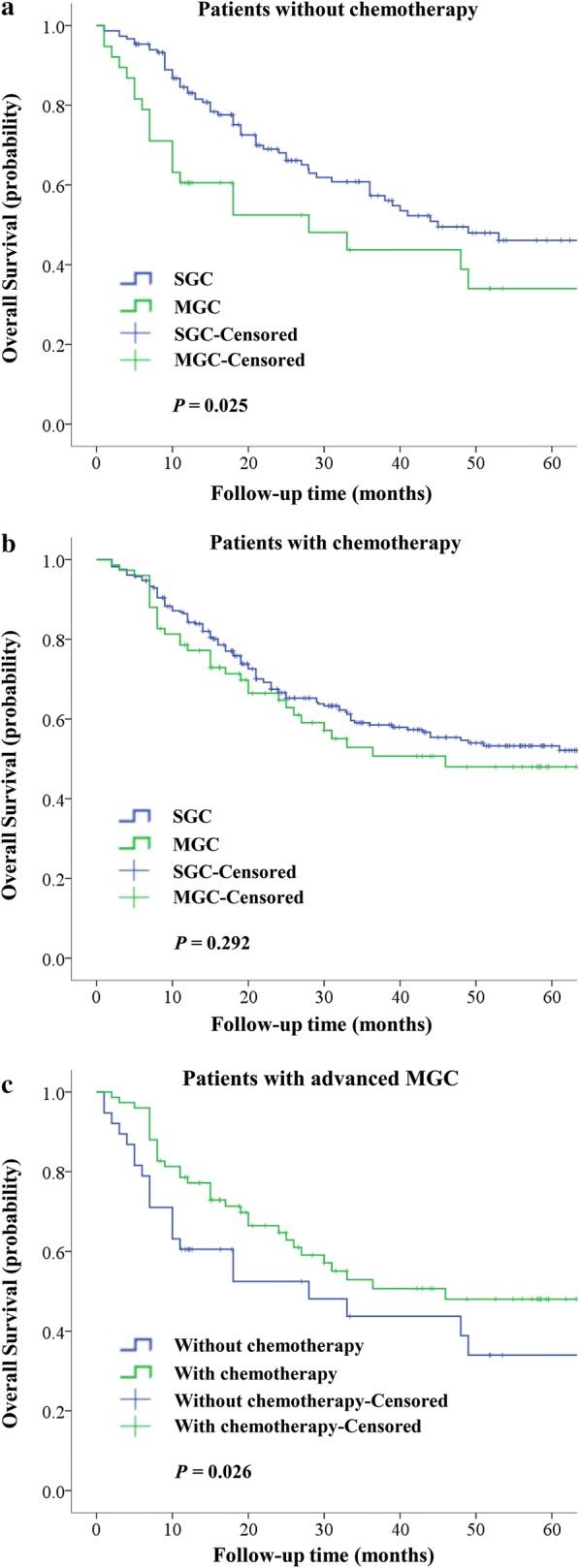
Impact of chemotherapy and advanced MGC on OS. **a** Impact of MGC on the OS of patients without chemotherapy. The 5-year OS rate of AGC patients who did not receive adjuvant chemotherapy was 43.4%. For the MGC and SGC groups, the 5-year OS rates were 34.0% and 46.1%, respectively (*P* = 0.025). **b** Impact of MGC on the OS of patients who had adjuvant chemotherapy. The 5-year OS rate of AGC patients who received adjuvant chemotherapy was 52.2%. For the MGC and SGC groups, the 5-year OS rates were 48.0% and 53.3%, respectively (*P* = 0.292). **c** Impact of adjuvant chemotherapy on the OS of patients with advanced MGC. The 5-year OS rate of patients with advanced MGC was 43.0%, of which the groups with and without adjuvant chemotherapy were 48.0% and 34.0%, respectively (*P* = 0.026). *MGC* multiple gastric cancers, *OS* overall survival

After stratification by adjuvant chemotherapy, the 5-year OS rate of advanced GC (AGC) patients who did not had adjuvant chemotherapy after surgery was found to be 43.4%, of which the 5-year OS rates of the MGC and SGC groups were 34.0% and 46.1%, respectively. This difference in 5-year OS was found to be statistically significant (*P* = 0.025, Fig. 4a). Regarding those AGC patients who received adjuvant chemotherapy, their overall 5-year survival rate was 52.2%, and the 5-year OS rates for the MGC and SGC groups were 48.0% and 53.3%, respectively, and no statistical difference in survival was observed (*P* = 0.292, Fig. 4b). Consequently, the 5-year OS rate of patients with advanced MGC was found to be 43.0%, of which the 5-year OS rates of those with adjuvant chemotherapy were significantly higher than those without adjuvant chemotherapy (48.0% vs. 34.0%, respectively; *P* = 0.026, Fig. 4c).

## Discussion

Our study demonstrated that the 5-year OS rate of patients with MGC was 48.1%, which was significantly poorer than those with SGC (58.7%) (*P* = 0.013) and that MGC was an independent risk factor for survival (Hazard ratio [HR] = 1.378, 95% confidence interval [CI] 1.061–1.870, *P* = 0.039). Further, our results identified that postoperative adjuvant chemotherapy could improve the 5-year OS rate of patients with advanced MGC.

Although most GCs consist of only one single lesion, MGCs are not uncommon. The incidence of MGC in our study was 3.2%. Some scholars believe that MGCs are a special type of GC, and there are differences in clinicopathological characteristics between MGC and SGC. Mitsudomi et al. found that MGCs were more common in elderly men and early GC [4]. In a study by Otsuji et al., they also found that patients with MGC and SGC had significant differences in tumor size, macroscopic types, depth of invasion, and the extent of lymph node dissection [6]. However, some scholars also believe that there are similarities between some of the clinicopathological features of MGC and SGC [3, 17–20]. In our study, MGC and SGC had no statistically significant differences in terms of demographic characteristics, such as age, sex, BMI, family history. Regarding postoperative pathology, MGC had deeper depths of invasion, greater number of lymph node metastases, and more advanced pathological stages, suggesting that MGCs may have a more aggressive nature in contrast to SGC.

Currently, there are few studies relating to the prognosis of patients with MGC, but those existing have shown contrasting results. Borie et al. investigated 199 cases of SGC and 33 cases of MGC. Their results showed that the 5-year OS rate of early MGC was greater than 90%, and was comparable to that of early SGC [8]. But in a study by Maeta et al. who analyzed 2241 cases of SGC and 164 cases of MGC, they found that the long-term prognosis of MGC cases was poorer than those of SGC in both stages I–II and III–IV [21]. However, these studies had significant differences in their investigated clinicopathological data, between the MGC and SGC groups, which may have led to the observed conflicting findings. In this study, we included a larger number of cases to analyze the clinicopathological differences between MGC and SGC cases, and we used a propensity score matching method to balance the differences between these two groups of patients to further explore the predictors associated with the prognosis for patients with MGC. Our results showed that the 5-year OS rate of MGCs was lower than that of SGCs, and that MGC was an independent predictor for postoperative OS. The reasons to why MGC patients have poor prognosis are still inconclusive. In a study which included 1606 patients who underwent gastrectomy with a follow-up for about 19 years postoperatively, the authors found that patients with MGC were more prone to metachronous cancers in the colon, urogenital system, and other organs than those with SGC [22]. Therefore, it is possible that patients with MGC may be more susceptible to canceration of other organs due to genetic susceptibility, leading to the poor long-term prognosis. In addition, Eom et al. reported that the prevalence of missed diagnosis of MGC by preoperative endoscopy was as high as 29.5% [7]. For such cases, if they did not undergo total gastrectomy, they could experience a higher risk postoperative tumor recurrence. Therefore, considerable awareness should be given to the possibility of multifocal cancer in the stomach. The preoperative, intraoperative, and postoperative examination of specimens should be strengthened and meticulously performed to avoid missed diagnoses.

In addition, we investigated the impact that different types of gastrectomy may have on the prognosis of MGC, but our results demonstrated that the type of gastrectomy was not an independent predictor of prognosis for patients with MGC (*P* = 0.114, Fig. 3d).

Further, this is the first study to have investigated the impact of chemotherapy on the prognosis of MGC. In this study, the 5-year OS rate of advanced MGC patients who received adjuvant chemotherapy was 48.0%. Most patients with advanced GC were recommended to receive 6 to 8 cycles of adjuvant chemotherapy. However, this depended on the patient’s performance status, major organ functions, severity of comorbidities and their willingness to comply with implement adjuvant chemotherapy. In clinical practice, we have observed that some proportion of our patients, especially those from rural areas or those who underwent surgery in the past decades, were reluctant to accept or comply with regular visits for postoperative adjuvant chemotherapy as compared to those being diagnosed in the recent years, and we hypothesize that these may have partly contributed to the cause of why some patients with AGC were not adjuvantly treated.

As shown in Fig. 4, our study found that for AGC patients without adjuvant chemotherapy, the 5-year OS rate of MGC was inferior than that of SGC (34.0% vs. 46.1%, *P* = 0.025), while for those who had adjuvant chemotherapy, the 5-year OS rates of patients with MGC and SGC were comparable (48.0% vs. 53.3%; *P* = 0.292). Further, we found that the 5-year OS rate of advanced MGC patients with adjuvant chemotherapy was significantly higher than those without adjuvant chemotherapy (48.0% vs. 34.0%; *P* = 0.026). The efficacy of adjuvant chemotherapy for AGC has been confirmed by multiple prospective studies. In multicenter studies from Japan and South Korea, the 5-year OS rate of patients receiving adjuvant chemotherapy after surgery was significantly higher than that of patients undergoing radical surgery alone [23, 24], and similar results were observed in western studies [25, 26]. In our study, chemotherapy has demonstrated significant survival benefits in patients with advanced MGC, however, the optimal regimen for such category of patients is yet to be determined.

Despite demonstrating the impact of adjuvant chemotherapy on the long-term survival of patients with MGC, the present study has several limitations that need to be addressed. First, the retrospective and non-randomized nature of this study makes it subjective to certain selection bias. Second, since our database only recorded whether the enrolled patients had or did not have adjuvant chemotherapy, data regarding the specific number of chemotherapy cycles were incomplete, and we believe this may have had some impacts on the results obtained, to some extent. Third, since we were unable to obtain accurate data on the type of cancer relapse (local recurrence, peritoneal metastasis, or distant metastasis) from our database, we could not analyze if there was a difference in the type of relapse between MGC and SGC. Fourth, due to the limited cases of MGC in our study, we did not further classify N3 stage as N3a and N3b. Despite these limitations, this study provided a reliable insight on the outcomes of patients with MGC and could be used as a preliminary basis for prospective multicenter studies on the treatment selection for this category of patients.

## Conclusions

In this study, by using propensity score matching analysis to balance the differences between cofounding variables, we have found that MGCs were an independent factor for survival and demonstrated worsen postoperative prognoses as compared to those with SGCs. However, the implementation of postoperative adjuvant chemotherapy to advanced MGC cases demonstrated significant improvement in their long-term survival and should be highly recommended.

## Abbreviations

GC: gastric cancer
MGC: multiple gastric cancer
SGC: solitary gastric cancer
AGC: advanced gastric cancer
OS: overall survival
AJCC: American Joint Committee on Cancer
UICC: Union for International Cancer Control
eLNs: examined lymph nodes
BMI: body mass index
ASA: American Society of Anesthesiologists
cT stage: clinical tumor stage
cN stage: clinical node stage
pT stage: pathological tumor stage
pN stage: pathological node stage
pTNM stage: pathological tumor-node-metastasis stage
WHO: World Health Organization
cN0: clinical node-negative
cN+: clinical node-positive
CT: computed tomography
HR: hazard ratio
CI: confidence interval
